# Reevaluating risk assessment in connective tissue disease-associated pulmonary arterial hypertension: The prognostic superiority of stroke volume index

**DOI:** 10.1515/rir-2025-0020

**Published:** 2025-10-04

**Authors:** Qingqing Cai, Huangshu Ye, Yixin Zhang, Jiayi Dai, Linwei Shan, Zhangdi Zhou, Dongyu Li, Ting Liu, Yanli Zhou, Fenghong Yuan, Xiaoxuan Sun

**Affiliations:** Department of Gerontology, Nanjing Hospital of Chinese Medicine Affiliated to Nanjing University of Chinese Medicine, Nanjing, Jiangsu Province, China; Department of Rheumatology, The First Affiliated Hospital with Nanjing Medical University, Nanjing, Jiangsu Province, China; Department of Cardiology, The First Affiliated Hospital with Nanjing Medical University, Nanjing, Jiangsu Province, China; Department of Rheumatology, The Affiliated Wuxi People’s Hospital of Nanjing Medical University, Wuxi, Jiangsu Province, China

**Keywords:** connective tissue diseases, pulmonary arterial hypertension, 2018 world symposia on pulmonary hypertension risk stratification, cardiac index, stroke volume index

## Abstract

**Objective:**

To evaluate the prognostic value of stroke volume index (SVI) compared to cardiac index (CI) in risk stratification and outcome prediction in connective tissue disease-associated pulmonary arterial hypertension (CTD-PAH).

**Methods:**

We performed a retrospective analysis of patients diagnosed with CTD-PAH through right heart catheterization (RHC) from two Chinese medical centers. This retrospective study analyzed 206 CTD-PAH patients, with risk stratification performed using the 2018 World Symposia on Pulmonary Hypertension (WSPH) framework. Restricted cubic splines (RCS) and log-rank tests were utilized to identify the optimal SVI cutof values for categorizing patients into low-, intermediate-, and high-risk groups. Kaplan-Meier (KM) curves were used to analyze survival rates and event-free survival. Receiver operating characteristic (ROC) analysis was used to assess the predictive accuracy of diferent models for prognostic outcomes.

**Results:**

SVI was categorized into low-risk (SVI ≥ 33.35 mL/m^2^), intermediate-risk (24.66 mL/m^2^≤ SVI < 33.35 mL/m^2^), and high-risk (SVI < 24.66 mL/m^2^) groups. Among the 206 CTD-PAH patients, 55 exhibited discrepancies in risk stratification between CI and SVI. SVI-based stratification provided more accurate risk categorization and demonstrated superior predictive value compared to CI, showing significant diferences in both survival and event-free survival rates across the groups.

**Conclusion:**

SVI enhances risk stratification and prognosis prediction in CTD-PAH by efectively distinguishing patients at higher risk for adverse outcomes.

## Introduction

Pulmonary arterial hypertension (PAH) is a life-threatening disease characterized by a progressive increase in pulmonary vascular resistance, ultimately leading to right heart failure and death.^[1]^ Among the various subtypes of PAH, connective tissue disease-associated PAH (CTD-PAH) accounts for approximately 20%–30% of cases and remains one of the most severe forms.^[2]^ Despite improvements in survival rates due to the introduction of PAH-specific therapies, CTD-PAH continues to carry a poor prognosis.^[3,4]^

Current guidelines for the management of PAH emphasize the importance of risk stratification in guiding therapy.^[5]^ The cardiac index (CI) is a key prognostic parameter used to assess mortality risk in PAH has been integrated into risk stratification models.^[6,7]^ However, recent evidence suggests that the prognostic value of CI may be inferior to other hemodynamic parameters, such as stroke volume index (SVI, defined as CI/HR [heart rate]) .^[8, 9, 10, 11, 12]^ Unlike CI (where CI= HR × SVI), SVI isolates per-beat output normalized to body size (SV/BSA). It therefore does not incorporate heart rate as a multiplicative component and is less confounded by compensatory tachycardia, providing a clearer representation of cardiac pump function. In the early stages of PAH, compensatory increases in heart rate can maintain CI within normal limits, even as cardiac function declines, thereby masking underlying dysfunction.^[13,14]^ Conversely, SVI, which reflects stroke volume adjusted for body surface area, shows significant reductions in such situations, offering a more sensitive marker of impaired cardiac performance.^[14,15]^

Based on these findings, we hypothesized that replacing CI with SVI in the 2018 World Symposia on Pulmonary Hypertension (WSPH) risk stratification models could improve survival phenotype discrimination in patients with CTD-PAH. To test this hypothesis, we conducted a study to evaluate whether SVI provides superior prognostic value compared to CI within current PAH risk stratification frameworks.

## Materials and Methods

### Study Design

We performed a retrospective analysis of patients diagnosed with CTD-PAH through right heart catheterization (RHC) between March 2013 and March 2024. Data were collected from two medical centers (The First Affiliated Hospital with Nanjing Medical University and Wuxi People’s Hospital). After applying the inclusion and exclusion criteria, a total of 206 patients with CTD-PAH were included in the final analysis.

This study has obtained ethics approval from the Institutional Review Board (2018-SR-333) and has been registered on ClinicalTrials. gov (NCT05980728).

### Inclusion and Exclusion Criteria

The inclusion criteria were as follows: (1) a confirmed diagnosis of CTD; (2) a confirmed diagnosis of PAH *via* RHC; and (3) age ≥18 years. The classification of connective tissue diseases (CTDs) in this study adhered to established criteria. Systemic lupus erythematosus (SLE) was classified according to the 2019 American College of Rheumatology/European League Against Rheumatism (ACR/EULAR) criteria.^[16]^ Primary Sjögren’s Syndrome (pSS) and systemic sclerosis (SSc) were classified based on the 2016 and 2013 ACR/EULAR criteria, respectively.^[17,18]^ The diagnosis of mixed connective tissue disease (MCTD) was determined using Sharp’s criteria.^[19]^ Patients meeting the diagnostic criteria for two or more CTDs were classified as having overlap syndrome (OS). Those presenting with characteristic CTD symptoms or signs, or high titers of non-organ-specific autoantibodies, but not fulfilling the diagnostic criteria for any specific CTD, were classified as having undifferentiated connective tissue disease (UCTD). The diagnostic criteria for PAH were as follows: mean pulmonary arterial pressure (mPAP) >20 mmHg (1 mmHg = 0.133 kPa) as measured by RHC, pulmonary arterial wedge pressure (PAWP) ≤15 mmHg, and pulmonary vascular resistance (PVR) > 2 Wood units 2022 European Society of Cardiology (ESC) and the European Respiratory Society (ERS) Guidelines.

Exclusion criteria included: (1) the presence of severe interstitial lung disease or chronic obstructive pulmonary disease (COPD), defined as forced vital capacity (FVC) < 60% of predicted value or forced expiratory volume in the first second (FEV1) < 70% of predicted value, or high-resolution chest computed tomography (CT) indicating severe interstitial fibrosis; (2) evidence of pulmonary thromboembolism, identified by radionuclide ventilation-perfusion scanning or CT pulmonary angiography; (3) liver cirrhosis with portal hypertension; (4) severe hematologic or metabolic diseases; (5) long-term use of drugs or toxins known to cause PAH, such as methamphetamine; (6) human immunodeficiency virus (HIV) infection.

### Data Collection and Outcomes

Baseline data for each patient included the following categories: (1) General characteristics: Age, sex, height, weight, cardiovascular risk factors, and the type of underlying CTD; (2) PAH assessment: levels of N-terminal pro b-type natriuretic peptide (NT-proBNP), World Health Organization (WHO) functional classification, 6-minute walk distance (6MWD), and parameters obtained from RHC; (3) Treatment regimen: medications for CTD, including glucocorticoids and immunosuppressants, as well as PAH-targeted therapies.

The primary endpoint of the study was all-cause mortality within 5 years. Our primary endpoint was all-cause mortality within 5 years, whereas the secondary endpoint was event-free survival, defined as the first occurrence of a clinical failure event (all-cause mortality or hospitalization for worsening PAH).

### Statistical Analysis

The distribution of continuous variables was expressed as mean ± standard deviation or interquartile range (Q1, Q3), while categorical variables were presented as frequencies and percentages. Kaplan-Meier curves were used to describe changes in overall survival and event-free survival, with the log-rank test employed to compare survival curves between groups. Restricted cubic spline (RCS) analysis was performed to examine the relationship between SVI and the risk of all-cause mortality. The SVI value corresponding to an HR of 1 was defined as the low-risk threshold, as SVI demonstrated a protective effect on prognosis when HR < 1. Additionally, a running log-rank test was conducted to identify the optimal high-risk cut-off point for SVI, which best discriminated patients with poor outcomes. SVI was then substituted for CI to reclassify patients according to the 2018 simplified risk stratification framework. The performance of the model was evaluated using Harrell’s concordance index (C-index) and time-dependent receiver operating characteristic (ROC) curves. Statistical significance was set at *P* < 0.05. All statistical analyses were conducted using SPSS version 26.0 (IBM Corp., Armonk, NY, USA) and the R statistical software package.

## Results

### Baseline Characteristics and Overall Survival of Patients with CTD-PAH

**Figure 1:**
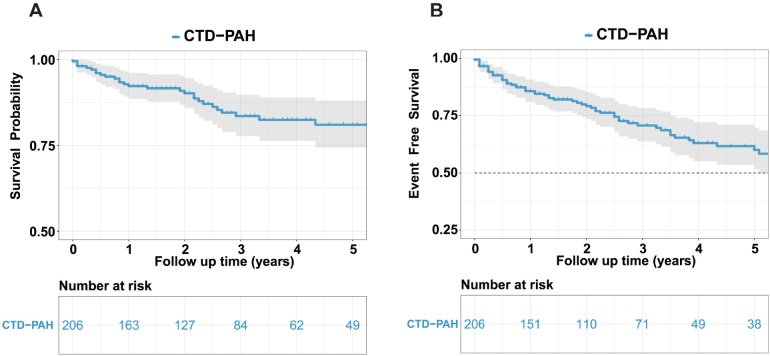
Kaplan–Meier Curves for overall survival and event-free survival in patients with CTD-PAH. Overall survival (A) and event-free survival (B) analysis in CTD-PAH patients. CTD, connective tissue disease-associated pulmonary arterial hypertension.

This retrospective study included 206 patients diagnosed with CTD-PAH. Baseline characteristics are summarized in Table 1. The median age of the cohort was 40.5 years (range: 31.2–55.0 years), with a predominance of female participants (94.66%). The underlying CTDs included SLE (32.52%), pSS (20.39%), SSc (8.74%), OS (22.33%), MCTD (6.31%), and UCTD (5.83%). The mean disease duration before enrollment was 12 months (range: 0.0–102.5 months). Additional baseline parameters, including demographic information, RHC parameters, and treatment therapies are detailed in Table 1–2. During a mean follow-up period of 46.98 ± 2.89 months, 28 patients died from all causes, with mortality distributed across subgroups as follows: SLE-PAH (*n* = 7), pSS-PAH (*n* = 7), SSc-PAH (*n* = 6), OS-PAH (*n* = 2), MCTD-PAH (*n* = 2), UCTD-PAH (*n* = 2), and other causes (*n* = 2). The cumulative overall survival rates at 1, 3, and 5 years were 92.3%, 83.5%, and 80.9%, respectively. A total of 57 patients experienced clinical failure events during follow-up, including SLE-PAH (*n* = 17), pSS-PAH (*n* = 15), SSc-PAH (*n* = 9), OS-PAH (*n* = 6), MCTD-PAH (*n* = 3), UCTD-PAH (*n* = 5), and other (*n* = 2). The corresponding event-free survival rates at 1, 3, and 5 years were 85.8%, 70.7%, and 60.0%, respectively (Figure 1 and Supplementary Figure S1).

**Table 1:** Baseline clinical characteristics of the whole study population

Variables	Total (*n* = 206)	SLE-PAH (*n* = 67)	pSS-PAH (*n* = 42)	SSc-PAH (*n* = 18)	OS-PAH^a^ (*n* = 46)	MCTD-PAH (*n* = 13)	UCTD-PAH (*n* = 12)	Others-PAH^b^ (*n* = 8)	Oveall *P*
Age, years	40.5 (31.2; 55.0)	33.0 (28.0; 41.0)	43.0 (34.0; 57.5)	57.5 (52.0; 63.8)	47.0 (36.0; 55.0)	45.0 (28.0; 57.0)	33.0 (28.8; 46.8)	48.5 (37.2; 61.0)	<0.001
Female, *n* (%)	195.0 (94.7)	65 (97.0)	40 (95.2)	14 (77.8)	44 (95.7)	13 (100)	12.0 (100.0)	7 (87.5)	0.087
BMI, kg/m^2^	21.3 (19.5; 24.0)	21.1 (19.7; 23.6)	22.3 (20.3; 24.0)	21.6 (20.4; 23.4)	20.8 (18.4; 22.4)	20.6 (19.0; 24.1)	23.3 (19.8; 24.3)	22.1 (21.2; 23.1)	0.535
Duration of CTD, months	12 .0 (0.0; 102.5)	36.0 (0.0; 138.0)	0.00 (0.0; 23.8)	49.0 (0.0; 104.0)	3.00 (0.0; 123.0)	0.0 (0.0; 87.0)	0.0 (0.0; 0.0)	94.5 (40.5; 154.0)	0.003
WHO FC, I-II	101 (49.0)	34 (50.7)	18 (42.9)	4 (22.2)	27 (58.7)	7 (53.8)	8.0 (66.7)	3 (37.5)	0.136
6MWD, m	440.5 (347.3; 500.5)	438.0 (356.5; 501.0)	443.0 (339.3; 473.3)	320.0 (266.5; 382.0)	468.5 (399.8; 532.5)	412.0 (389.5; 497.0)	455.0 (314.3; 495.0)	420.0 (389.0; 444.0)	0.027
NT-proBNP, pg/mL	699.0 (195.0; 2304.5)	598.0 (218.3; 3310.0)	1011.3 (225.3; 2797.0)	622.0 (172.1; 3880.0)	622.9 (192.5; 1849.0)	905.3 (370.1; 1690.0)	329.3 (179.5; 615.5)	1045.0 (275.7; 1856.0)	0.759
Heart Rate, min^-1^ RHC parameters	84 (76; 95)	88 (78; 100)	83 (73; 94)	84 (80; 91)	82 (75; 92)	89 (82; 95)	80 (74; 91)	85 (78; 89)	0.303
mPAP, mmHg	41.00 (33.0, 51.0)	41.0 (33.5; 51.5)	41.5 (35.2; 50.8)	41.5 (35.5; 48.0)	37.5 (32.0; 46.0)	39.0 (32.0; 43.0)	47.5 (42.5; 52.0)	41.0 (31.2; 50.5)	0.341
PVR, wood units	7.5 (4.8;11.6)	7.5 (4.7;13.4)	9.4 (5.0; 12.4)	8.3 (5.9; 9.4)	6.4 (4.2; 8.4)	7.1 (5.1; 8.4)	7.4 (6.8; 14.2)	10.1 (6.2;12.4)	0.351
PAWP, mmHg	8.0 (5.0; 11.0)	8.0 (5.0;11.2)	9.0 (5.0; 11.0)	9.50 (6.0; 13.0)	7.0 (6.0; 9.0)	7.0 (6.0; 9.0)	9.0 (7.0; 13.2)	7.0 (5.8; 9.5)	0.630
mRAP, mmHg	5.0 (3.0; 8.0)	6.0 (3.0; 8.0)	4.0 (2.0; 8.8)	5.00 (3.0; 9.0)	4.50 (3.0; 7.0)	4.0 (2.0; 8.0)	5.0 (3.8; 7.3)	4.0 (2.8;8.0)	0.956
SvO2, %	65.0 (59.0; 70.0)	64.0 (59.0; 71.0)	64.0 (58.0; 69.0)	68.5 (57.2; 71.6)	65.0 (61.0; 70.5)	65.5 (63.0;68.0)	66.0 (62.0; 70.5)	60.5 (54.2; 67.2)	0.778
CI, L/min/m^2^	2.8 (2.2; 3.4)	3.0 (2.2; 3.7)	2.6 (2.1; 2.9)	2.7 (2.3; 3.0)	3.0 (2.50;3.61)	2.9 (2.5; 3.5)	2.56 (2.2; 3.1)	2.3 (1.4; 3.1)	0.053
SVI, mL/m^2^	33.4 (24.9; 41.0)	34.0 (24.9; 40.3)	31.0 (23.8; 39.2)	31.9 (24.4; 35.7)	36.3 (29.0; 46.7)	32.9 (30.4;36.6)	29.9 (24.1; 41.7)	26.4 (19.8; 35.7)	0.094
Binary therapy^c^, *n* (%)	128 (62.1)	51 (76.1)	26 (61.9)	11 (61.1)	24 (52.2)	8 (61.5)	5 (41.7)	3 (37.5)	0.058
CTD treatment, *n* (%)									
Glucocorticoid^d^	183 (88.8)	67 (100.0)	37 (88.1)	14 (77.8)	40 (87.0)	13 (100.0)	8 (66.7)	4 (50.0)	<0.001
Low dose	79 (38.3)	26 (38.8)	17 (40.5)	9 (50.0)	16 (34.8)	5 (38.5)	5 (41.7)	1 (12.5)	-
Medium dose	76 (36.9)	25 (37.3)	15 (35.7)	5 (27.8)	17 (37.0)	8 (61.5)	3 (25.0)	3 (37.5)	-
High dose	28 (13.6)	16 (23.9)	5 (11.9)	0 (0.0)	7 (15.2)	0 (0.0)	0 (0.0)	0 (0.0)	-
Hydroxychloroquine	151 (73.3)	56 (83.6)	32 (76.2)	8 (44.4)	33 (71.7)	8 (61.5)	9 (75.0)	5 (62.5)	0.041
Immunosuppressant	138 (67.0)	52 (77.6)	28 (66.7)	14 (77.8)	25 (54.3)	9 (69.2)	5 (41.7)	5 (62.5)	0.077
CTX	89 (43.2)	30 (44.8)	22 (52.4)	7 (38.9)	19 (41.3)	2 (15.4)	5 (41.7)	4 (50.0)	0.413
MMF	22 (10.7)	11 (16.4)	3 (7.1)	3 (16.7)	2 (4.4)	3 (23.1)	0 (0.0)	0 (0.0)	0.132
AZA	7 (3.4)	3 (4.5)	0 (0.0)	2 (11.1)	0 (0.0)	2 (15.4)	0 (0.0)	0 (0.0)	0.041
CNI	13 (6.3)	6 (9.0)	3 (7.1)	1 (5.6)	2 (4.4)	1 (7.7)	0 (0.0)	0 (0.0)	0.958
IIT^e^	132 (64.1)	52 (77.6)	27 (64.3)	11 (61.1)	24 (52.2)	9 (69.2)	5 (41.7)	4 (50.0)	0.058
PAH targeted treatment^f^, n (%)									0.267
None	8 (3.9)	2 (3.0)	3 (7.1)	0 (0.0)	0 (0.0)	2 (15.4)	0 (0.0)	1 (12.5)	-
Monotherapy	57 (27.7)	22 (32.8)	11 (26.2)	5 (27.8)	12 (26.1)	3 (23.1)	1 (8.3)	3 (37.5)	-
Combination therapy	141 (68.5)	43 (64.2)	28 (66.7)	13 (72.2)	34 (73.9)	8 (61.5)	11 (91.7)	4 (50.0)	-

^a^Detailed diagnostic composition for OS patients is provided in Supplementary Table S1; ^b^Other underlying aetiologies of CTD-PAH including: rheumatoid arthritis (n = 6) and ankylosing spondylitis (*n* = 2); ^c^Binary therapy was defined as receiving both PAH-targeted therapy and CTD therapy simultaneously; ^d^Low dose Equivalent to prednisone >0.5 mg/kg/d; medium dose Equivalent to prednisone 0.5–1 mg/kg/d; High dose equivalent to prednisone ≥1 mg/kg/d; ^e^IIT, intensive immunosuppressive therapy, receiving both glucocorticoids and immunosuppressant; ^f^PAH-targeted therapy included monotherapy with PCA, PDEi5, ERA, IP receptor agonists and a combination of the above drugs on different pathways. Abbreviation: OS, overlap syndrome; BMI, body mass index; CTD, connective tissue disease; WHO FC, World Health Organization functional class; 6MWD, 6-minute walk distance; RHC, right heartcatheterization; mPAP, mean pulmonary arterial pressure; PVR, pulmonary vascular resistance; PAWP, pulmonary arterial wedge pressure; mRAP, mean right atrial pressure; CI, cardiac index; SvO2, mixed venous oxygen saturation; SVI, stroke volume index; CTX, Cyclophosphamide; MMF, Mycophenolate Mofetil; AZA, Azathioprine; CNI, Calcineurin inhibitor.

**Table 2:** Baseline clinical characteristics of the 55 patients

Variables	Low risk		*P1*	Intermediate risk		*P2*	High risk		*P3*
	Using CI (*n* = 11)	Using SVI (*n* = 5)		Using CI (*n* = 32)	Using SVI (*n* = 30)		Using CI (*n* = 12)	Using SVI (*n* = 20)	
Age, years	34.0 (32.5; 42.0)	37.0 (34.0; 42.0)	0.774	40.0 (27.8; 59.3)	34.50 (29.3; 49.8)	0.526	55.5 (44.8; 61.5)	55.5 (44.8; 63.0)	0.725
Female, *n* (%)	11 (100.0)	5 (100.0)	>0.999	30 (93.8)	29 (96.7)	>0.999	11 (91.7)	18 (90.0)	>0.999
BMI, kg/m^2^	21.6 ± 2.8	21.1 ± 2.683	0.707	20.7 (18.5;22.9)	20.8 (18.4; 23.0)	0.911	19.2 (17.9; 23.3)	20.7 (18.7; 24.0)	0.651
Underlying CTD, *n* (%)			>0.999			0.992			0.991
SLE	6 (54.5)	3 (60.0)	-	7 (21.9)	8 (26.7)	-	3 (25.0)	5 (25.0)	-
pSS	3 (27.3)	1 (20.0)	-	8 (25.0)	6 (20.0)	-	3 (25.0)	7 (35.0)	-
SSc	0 (0.0)	0 (0.0)	-	7 (21.9)	6 (20.0)	-	3 (25.0)	4 (20.0)	-
OS	2(18.2)	1 (20.0)	-	3 (9.4)	4 (13.3)	-	1 (8.33)	1 (5.0)	-
MCTD	0 (0.0)	0 (0.0)	-	3 (9.4)	3 (10.)	-	1 (8.3)	1 (5.0)	-
UCTD	0 (0.0)	0 (0.0)	-	4 (12.5)	3 (10.0)	-	0 (0.0)	1 (5.0)	-
RA	0 (0.0)	0 (0.0)	-	0 (0.0)	0 (0.0)	-	1 (8.3)	1 (5.0)	-
Duration of CTD, months	90.0 (23.0; 145.0)	90.0 (44.0; 167.0)	0.776	0.0 (0.0; 67.8)	0.0 (0.0; 107.3)		0.0 (0.0; 23.5)	10.0 (0.0; 30.8)	0.465
WHO FC, I-II	10 (90.9)	5 (100.0)	>0.999	13 (40.6)	16 (53.3)	0.455	1 (8.3)	3 (15.0)	>0.999
6MWD, m	504.5 (482.0; 518.3)	513.5 (505.8; 545.0)	0.320	409.0 (324.3; 492.5)	454.0 (378.0; 505.5)	0.262	368.0 (345.0; 459.0)	365.0 (300.0; 459.0)	0.639
NT-proBNP, pg/mL	201.7 (136.5; 398.8)	193.0 (159.2; 201.7)	0.570	1797.0 (370.5; 2802.0)	622.0 (336.3; 2315.0)	0.166	3996.0 (1553.8; 4488.3)	3971.5 (1748.0; 4327.0)	0.799
Heart Rate, min^-1^	91 (85; 95)	94 (71; 95)	0.955	93 (84; 100)	92 (84; 99)	0.558	94 (91; 104)	95 (91; 103)	0.969
RHC parameters									
mPAP, mmHg	46.7 ± 11.1	38.6 ± 8.4	0.136	41.5 (37.0; 50.3)	43.5 (37.0; 50.8)	0.810	51.3 ± 10.4	50.6 ± 10.6	0.867
PVR, wood units	9.4 ± 3.0	7.5 ± 2.2	0.166	9.4 ± 3.9	8.9 ± 3.5	0.626	11.3 (8.1; 18.1)	11.7 (9.4; 17.2)	0.887
PAWP, mmHg	7.5 ± 3.2	7.8 ± 3.6	0.860	10.0 (6.0; 13.0)	8.0 (6.0;12.0)	0.689	10.5 ± 2.8	10.0 ± 3.1	0.631
mRAP, mmHg	3.4 ± 1.9	3.6 ± 1.9	0.826	7.0 (3.0; 8.3)	5.0 (3.0; 8.0)	0.523	8.0 (3.0; 12.0)	6.5 (3.0; 11.3)	0.723
SvO2, %	67.2 ± 5.0	64.5 ± 6.5	0.487	64.5 (63.0; 70.0)	66.1 (64.0; 70.0)	0.265	50.5 ± 6.4	55.3 ± 9.2	0.096
CI, L/min/m^2^	2.7 ± 0.3	2.7 ± 0.3	0.926	2.6 (2.2; 2.8)	2.7 (2.6; 2.8)	0.171	2.2 (2.0; 2.6)	2.2 (2.0; 2.3)	0.784
SVI, mL/m^2^	30.9 ± 4.0	32.6 ± 4.6	0.484	27.1 (24.0; 31.0)	29.9 (26.1; 32.2)	0.135	22.7 (21.3; 28.2)	22.7 (20.9; 24.3)	0.846
Binary therapy^a^, *n* (%)	7 (63.6)	3 (60.0)	>0.999	18 (56.3)	18 (60.0)	0.615	8 (66.7)	12 (60.0)	>0.999
CTD treatment, *n* (%)									
Glucocorticoid^b^	11	5	>0.999	26 (81.3)	26 (86.7)	0.733	10 (83.3)	16 (80.0)	>0.999
Low dose	8 (72.7)	3 (60.0)	-	6 (18.8)	4 (13.3)	-	5 (41.7)	9 (45.0)	-
Medium dose	2 (18.2)	1 (20.0)	-	9 (28.1)	10 (33.3)	-	4 (33.3)	4 (20.0)	-
High dose	1 (9.1)	1 (20.0)	-	13 (40.6)	14 (46.7)	-	1 (8.3)	3 (15.0)	-
Hydroxychloroquine	10 (90.9)	5 (100.0)	>0.999	17 (53.1)	19 (63.3)	0.578	8 (66.7)	11 (55.0)	0.713
Immunosuppressant	8 (72.7)	4(75.0)	>0.999	21 (65.6)	21 (70.0)	0.923	9 (75.0)	13 (65.0)	0.703
CTX	6 (54.5)	3 (60.0)	>0.999	14 (43.8)	15 (50.0)	0.622	7 (58.3)	9 (45.0)	0.716
MMF	1 (9.1)	0 (0.0)	>0.999	4 (12.5)	4 (13.3)	>0.999	2 (16.7)	3 (15.0)	>0.999
AZA	1 (9.1)	1 (20.0)	>0.999	1 (3.1)	0 (0.0)	>0.999	0 (0.0)	1 (5.0)	>0.999
CNI	0 (0.0)	0 (0.0)	-	1 (3.1)	1 (3.3)	>0.999	0 (0.0)	0 (0.0)	-
IIT^c^	8 (72.7)	4 (80.0)		19 (59.4)	19 (63.3)	0.953	8 (66.7)	12 (60.0)	>0.999
PAH targeted treatmentd *n* (%)			>0.999			0.615			0.303
None	1 (9.1)	1 (20.0)	-	2 (6.3)	1 (3.3)	-	1 (8.3)	2 (10.0)	-
Monotherapy	1 (9.1)	0 (0.0)	-	11 (34.4)	8 (26.7)	-	0 (0.0)	4 (20.0)	-
Combination therapy	9 (81.8)	4 (80.0)	-	19 (59.4)	21 (70.0)	-	11 (91.7)	14 (70.0)	-

a Binary therapy was defined as receiving both PAH-targeted therapy and CTD therapy simultaneously; ^b^Low dose Equivalent to prednisone <0.5 mg/kg/d; medium dose Equivalent to prednisone 0.5–1 mg/kg/d; High dose equivalent to prednisone ≥1 mg/kg/d; ^c^IIT, intensive immunosuppressive therapy, receiving both glucocorticoids and immunosuppressant; ^d^PAH-targeted therapy included monotherapy with PCA, PDEi5, ERA, IP receptor agonists and a combination of the above drugs on different pathways. OS, overlap syndrome; BMI, body mass index; CTD, connective tissue disease; WHO FC, World Health Organization functional class; 6MWD, 6-minute walk distance; RHC, right heartcatheterization; mPAP, mean pulmonary arterial pressure; PVR, pulmonary vascular resistance; PAWP, pulmonary arterial wedge pressure; mRAP, mean right atrial pressure; CI, cardiac index; SvO2, mixed venous oxygen saturation; SVI, stroke volume index; CTX, Cyclophosphamide; MMF, Mycophenolate Mofetil; AZA, Azathioprine; CNI, Calcineurin inhibitor.

### Defining SVI Cut-offs for Mortality Risk in CTD-PAH

**Figure 2:**
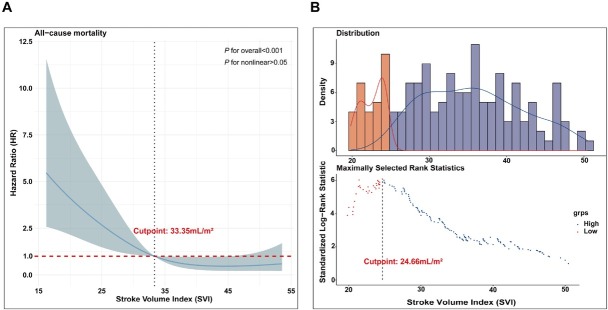
Defining SVI cut-offs for mortality risk in CTD-PAH. RCS analysis to reveal a linear relationship between SVI and mortality risk in CTD-PAH patients (A); A running log-rank test was performed on the survival curves of CTD-PAH patients (B). SVI, stroke volume index; CTD, connective tissue disease-associated pulmonary arterial hypertension; RCS, restricted cubic splines.

In this study, RCS were employed to investigate the relationship between SVI and mortality risk in patients with CTD-PAH. The analysis revealed a linear association between SVI and mortality risk (overall *P* < 0.001; non-linear *P* > 0.05), indicating that a decrease in SVI is associated with an increased risk of mortality. Specifically, when the HR was equal to 1, the corresponding SVI value was 33.35 mL/m^2^, establishing this as the cutoff for low risk (Figure 2A). Subsequently, a running log-rank test was conducted on the survival curves of CTD-PAH patients to determine the optimal SVI cut-off value. The results identified an SVI of 24.66 mL/m^2^as the point where the survival curves exhibited the most significant separation (Figure 2B). Therefore, this threshold was defined as the high-risk cuto-off for distinguishing mortality risk in CTD-PAH patients.

### Prognostic Value of SVI in CTD-PAH Risk Stratification

**Figure 3:**
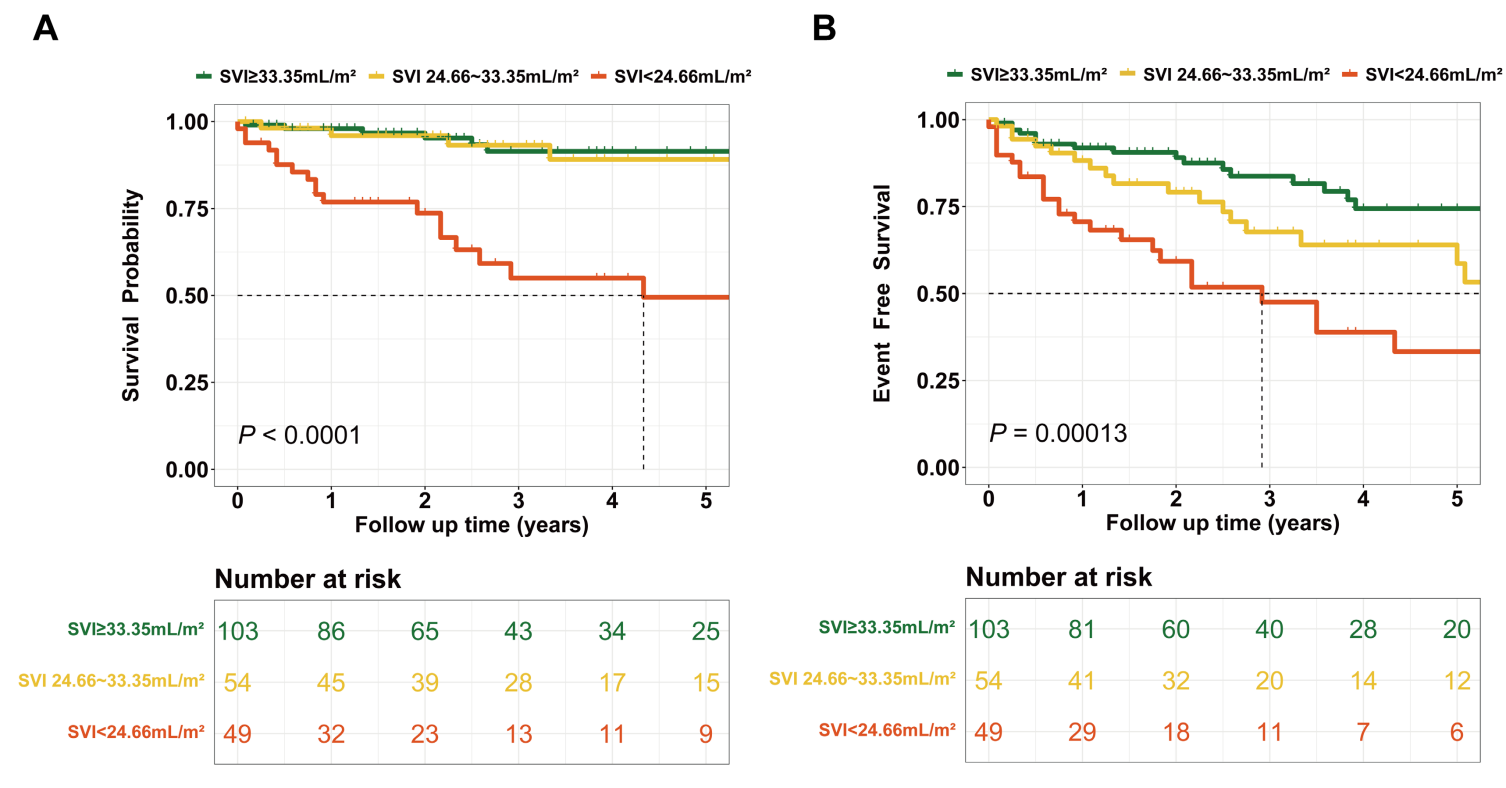
Prognostic value of SVI in CTD-PAH risk stratification. Overall survival (A) and event-free survival (B) analysis curves for CTD-PAH patients stratified by SVI levels. SVI, stroke volume index; CTD, connective tissue disease-associated pulmonary arterial hypertension.

Patients with CTD-PAH were categorized into three risk groups based on SVI thresholds of 33.35 mL/m^2^and 24.66 mL/m^2^: (1) Low-risk group: SVI ≥ 33.35 mL/m^2^; (2) Intermediate-risk group: 24.66 mL/m^2^≤ SVI < 33.35 mL/m^2^; and (3) High-risk group: SVI < 24.66 mL/m^2^. During follow-up, 6 patients in the low-risk group died, and 17 experienced clinical failure (Figure 3A-B). The cumulative survival rates at 1, 3, and 5 years were 98.0%, 91.4%, and 91.4%, respectively, while the event-free survival rates were 91.9%, 83.7%, and 74.4%. In the intermediate-risk group, 4 patients died, and 16 experienced clinical failure. The cumulative survival rates at 1, 3, and 5 years were 95.9%, 93.2%, and 89.1%, respectively, with event-free survival rates of 88.2%, 67.7%, and 58.6%. In the high-risk group, 18 patients died, and 24 experienced clinical failure. The cumulative survival rates at 1, 3, and 5 years were 76.9%, 55.0%, and 49.5%, respectively, while the event-free survival rates were 70.7%, 47.5%, and 33.3%. These findings highlight significant differences in long-term survival and event-free survival outcomes across the risk groups. As SVI decreases, both survival and event-free survival rates decline, underscoring the potential of SVI as an important prognostic marker in CTD-PAH management.

### Comparative Analysis of CI and SVI for Risk Stratification and Prognostic Accuracy in 2018 WSPH Assessment

**Figure 4:**
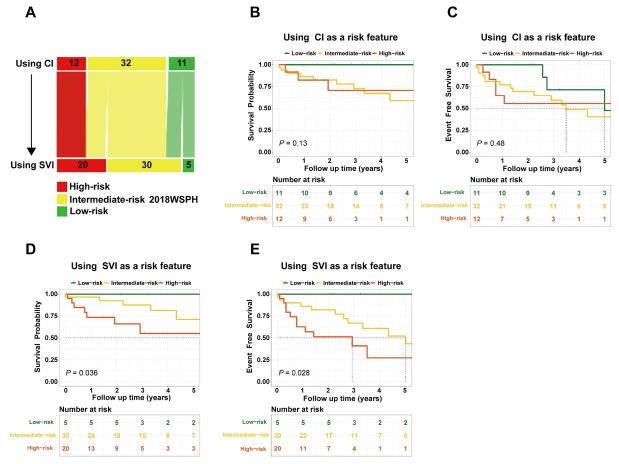
SVI was used as an alternative to CI in the 2018 WSPH risk assessment (A); Overall survival and event-free survival rates in the 2018 WSPH risk stratification based on CI (B-C) and SVI (D-E). CI, cardiac index; SVI, stroke volume index; WSPH, World Symposia on Pulmonary Hypertension.

Among the overall cohort (*n* = 206), 55 patients exhibited discordant CI-based *versus* SVI-based WSPH risk classifications and were included in the comparative analyse. The results indicated that 5 low-risk patients were reclassified to the intermediate-risk category, while 8 intermediate-risk patients were upgraded to high-risk (Figure 4A). In the 2018 WSPH risk stratification based on CI (Figure 4B-C), 11 patients were classified as low risk (1-, 3-, and 5-year survival and event-free survival rates were 100%, 100%, 100%, and 100%, 71.4%, 47.6%, respectively); 32 as intermediate risk (1-, 3-, and 5-year survival rates and event-free survival rates were 86.5%, 72.8%, 58.8%, and 77.1%, 59.4%, 40.5%, respectively); and 12 as high risk (1-, 3-, and 5-year survival and event-free survival rates were 82.5%, 70.7%, 70.7%, and 64.8%, 55.6%, 55.6%, respectively). No significant differences were observed in survival rates or event-free survival rates among the three groups. In the 2018 WSPH risk stratification based on SVI (Figure 4D-E), 5 patients were classified as low risk (1-, 3-, and 5-year survival and event-free survival rates were all 100%); 30 as intermediate risk (1-, 3-, and 5-year survival and event-free survival rates were 96.7%, 87.6%, 71.2%, and 86.1%, 66.9%, 43.4%, respectively); and 20 as high risk (1-, 3-, and 5-year survival and event-free survival rates were 73.4%, 55.1%, 36.7%, and 62.7%, 41.0%, 27.4%, respectively). Significant differences were found between the three groups. Statistically significant differences were observed between the three groups. SVI demonstrated a tendency to outperform CI in discriminating patient outcomes.

### Comparative Predictive Performance of CI- and SVI-Based 2018 WSPH Risk Stratification Models in CTD-PAH

**Figure 5:**
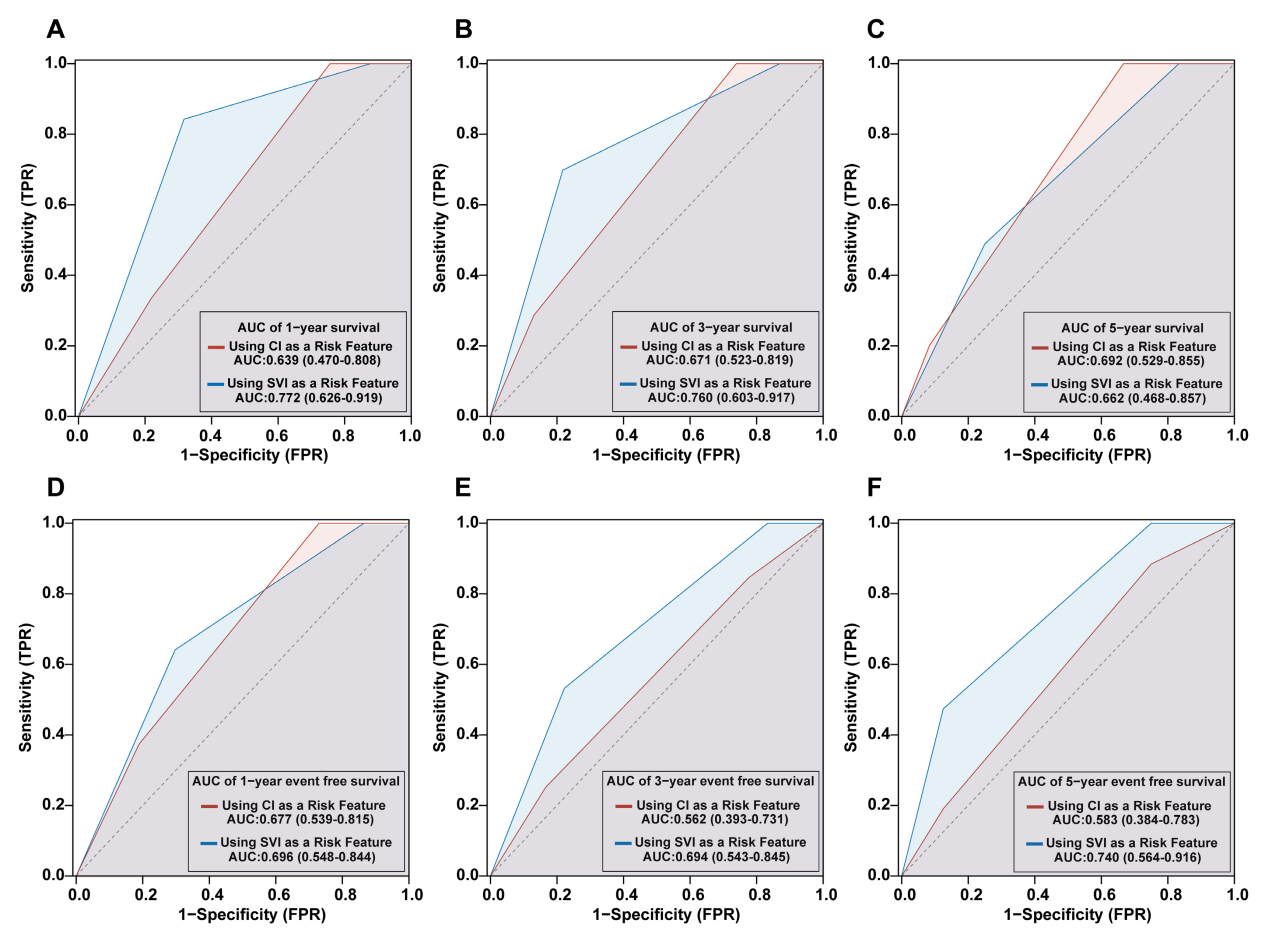
Comparative predictive performance of CI- and SVI-Based 2018 WSPH risk stratification models in CTD-PAH: Survival (A-C) and event-free survival (D-F) outcomes. CI, cardiac index; SVI, stroke volume index; WSPH, World Symposia on Pulmonary Hypertension; CTD, connective tissue disease-associated pulmonary arterial hypertension.

Figure 5 depicts the time-dependent ROC analysis for predicting adverse outcomes in CTD-PAH using the 2018 WSPH risk stratification based on CI *versus* SVI. For all-cause mortality, CI-based stratification yielded AUCs of 0.639 (95% CI, 0.470–0.808), 0.671 (0.523–0.819), and 0.692 (0.529–0.855) at 1, 3, and 5 years, respectively; for event-free survival, AUCs were 0.677 (0.539–0.815), 0.562 (0.393–0.731), and 0.583 (0.384–0.783)(Figure 5A–C). In contrast, SVI-based stratification produced AUCs of 0.772 (0.626–0.919), 0.760 (0.603–0.917), and 0.662 (0.468–0.857) for 1-, 3-, and 5-year mortality, and 0.696 (0.548–0.844), 0.694 (0.543–0.845), and 0.740 (0.564–0.916) for event-free survival (Figure 5D–F). Overall, SVI-based stratification demonstrated higher discrimination than CI-based stratification across most time points.

## Discussion

SVI, which reflects per-beat stroke volume normalized to body surface area (SV/BSA), does not include heart rate as a multiplicative factor (SVI = CI/HR) and is therefore less influenced by changes in heart rate than CI. By definition, CI = HR×SVI; thus increases in HR can maintain CI despite falling SVI, potentially masking early ventricular dysfunction. In this study, our key findings include: (1) SVI as an important prognostic marker in CTD-PAH management; (2) SVI demonstrated superior prognostic value over CI in PAH risk stratification by effectively differentiating survival outcomes across risk groups; (3) SVI-based stratification outperformed CI-based models in predicting survival and event-free outcomes in CTD-PAH patients.

SVI plays a pivotal role in the prognostic evaluation of PAH, offering critical insights into right ventricular function and its impact on disease progression.^[8, 9, 10, 11]^ As a measure of the volume of blood ejected by the heart per heartbeat, normalized to body surface area, SVI reflects both right ventricular contractility and afterload, which are key determinants of PAH outcomes.^[20, 21, 22]^ Recent studies have highlighted the importance of SVI in stratifying PAH patients into risk categories and predicting long-term survival and event-free survival.^[23]^ In this study, we used RCS and log-rank tests to stratify SVI into low-, intermediate-, and high-risk categories, providing an effective framework for predicting adverse outcomes in patients with CTD-PAH. SVI serves as a direct marker of right heart performance, correlating with right ventricular-arterial coupling—a fundamental concept in understanding PAH pathophysiology.^[24]^ Impaired SVI often signals advanced disease and an increased likelihood of adverse outcomes.^[25, 26, 27]^

Although the 2018 WSPH guidelines predominantly rely on CI for risk assessment, recent studies have highlighted its limitations in fully capturing the intricate hemodynamic changes that impact patient outcomes.^[28]^ In contrast, SVI, which quantifies the amount of blood ejected per heartbeat adjusted for body surface area, provides a direct reflection of right ventricular function—an essential determinant of prognosis in PAH.^[20, 21, 22]^ In our cohort, among patients with different risk stratifications of CI and SVI, SVI-based stratification more effectively identified CTD-PAH patients at higher risk for adverse outcomes. Compared to CI, SVI offers distinct advantages in assessing right heart function in patients with PAH, particularly by accounting for the compensatory effects of heart rate. While CI is a widely used measure of cardiac output, it does not differentiate between the contributions of stroke volume and heart rate to overall cardiac performance.^[29]^ This limitation is particularly relevant in PAH, where heart rate can significantly compensate for impaired stroke volume, masking subtle right ventricular dysfunction.^[12]^ In contrast, SVI, by normalizing stroke volume to body surface area, provides a more accurate reflection of right ventricular performance, independent of heart rate fluctuations.^[9,15]^ In the context of PAH, the heart often compensates for increasing PVR by increasing heart rate, a phenomenon that may obscure early signs of right heart failure when only CI is used. SVI, by focusing on stroke volume, can better detect early right ventricular dysfunction, as it is less influenced by the heart rate compensatory mechanism.^[14]^ This makes SVI particularly valuable in identifying patients with PAH who may have impaired right ventricular function but normal or elevated heart rates, which could be overlooked using CI alone. Moreover, previous study has demonstrated that SVI-based risk stratification provides a more accurate prediction of adverse outcomes in PAH patients, outperforming CI in identifying high-risk patients.^[12]^ This enhanced sensitivity in detecting early right ventricular dysfunction, independent of compensatory heart rate increases, makes SVI an essential tool for prognosis in SSc-PAH.^[8,10,12]^ By more precisely reflecting right ventricular performance, SVI allows for better risk stratification and treatment planning, particularly in cases where heart rate compensation may otherwise mask underlying hemodynamic abnormalities.

Beyond minimizing heart-rate confounding, SVI provides physiological insight into right ventricular-pulmonary artery (RV–PA) coupling. Coupling is defined by Ees/Ea, where Ea in the pulmonary circulation can be approximated as pressure/stroke volume; therefore, a lower SVI (SV/BSA) at a given pulmonary pressure implies higher Ea and worse RV– PA coupling. In parallel, pulmonary arterial compliance (PAC) is commonly estimated as SV/pulse pressure; reductions in SVI are consistent with diminished PAC and increased pulsatile load. Collectively, these relations explain why SVI declines as afterload rises and RV contractile–arterial matching deteriorates, complementing non-invasive coupling surrogates such as tricuspid annular plane systolic excursion (TAPSE) / pulmonary arterial systolic pressure (PASP).^[30]^

Despite the strengths of our study, several limitations should be noted. First, the relatively small sample size may limit the generalizability of our findings. Second, the retrospective nature of the study introduces potential selection bias. Thirdiy, we now explicitly acknowledge that combining heterogeneous CTD-PAH etiologies in a single analysis is a limitation and may dilute subtype-specific effects; the exploratory subgroup analyses were underpowered, and residual confounding by subtype-related factors (*e.g*., disease duration, immunotherapy, right-heart afterload profile) may persist. Future research should include larger, multi-center cohorts and prospective designs to validate these findings.

## Conclusions

This study demonstrates that SVI offers a more accurate assessment of right ventricular function than CI in CTD-PAH patients. SVI-based risk stratification better identifies patients at higher risk for adverse outcomes, detecting subtle right ventricular dysfunction independent of heart rate compensation. Our findings suggest that SVI enhances existing risk models, improves prognostication, and could serve as a valuable tool for personalized treatment planning in CTD-PAH. Further studies are needed to validate its role in routine clinical practice and across diverse PAH populations.

## Supplementary Material

Supplementary Material Details
