# Supplementary material for: Reevaluating risk assessment in connective tissue disease-associated pulmonary arterial hypertension: The prognostic superiority of stroke volume index
Source: Rheumatol Immunol Res. 2025 Oct 4;6(3):168–78. doi: 10.1515/rir-2025-0020 (PMC12495985; doi:10.1515/rir-2025-0020)
Supplement: Supplementary file 1 — Supplementary Material Details [file rir-2025-0020_sm.pdf]

Supplementary material

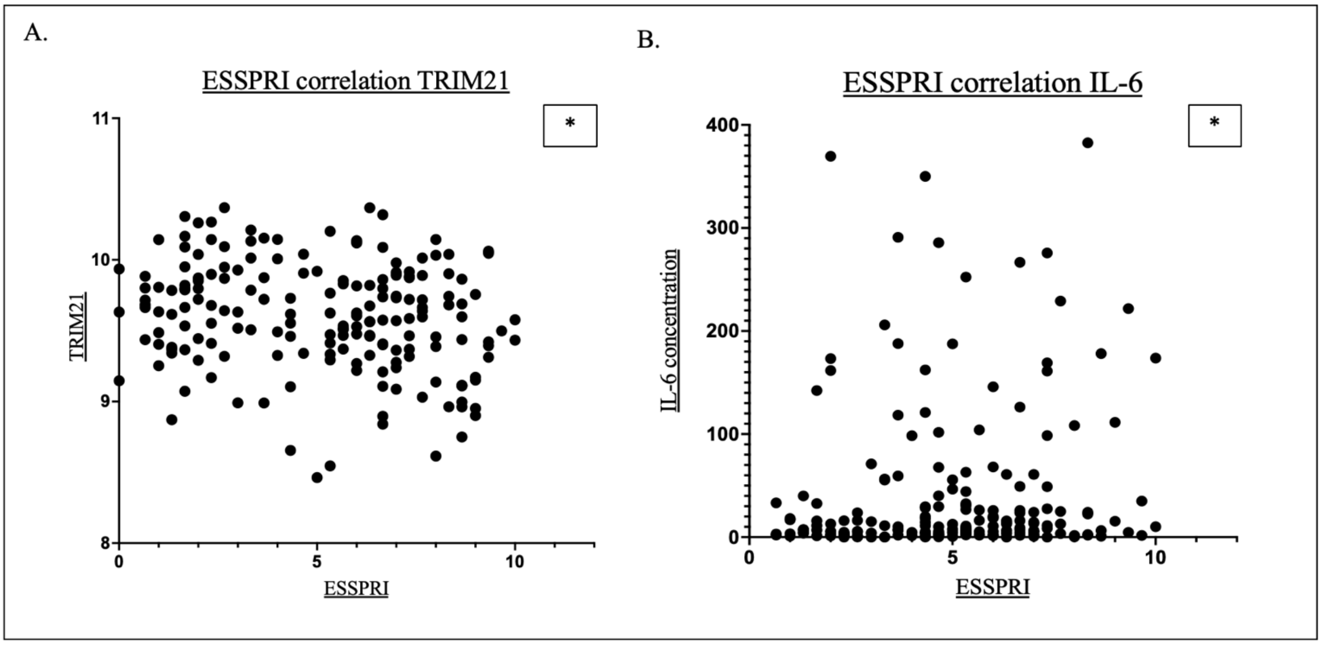

Supplementary Figure 1. Scatterplots showing the correlation between A) TRIM21 expression and ESSPRI symptom score B) IL-6 concentration and ESSPRI symptom score.

The correlation between total ESSPRI score and TRIM21 expression had a weak negative correlation coefficient of -0.1879, Fig. 5A). (*P* = 0.0106).

In contrast, there is a weak positive correlation between total ESSPRI score and IL-6 (correlation coefficient of 0.1563, *P* = 0.0299) (Fig. 5B).

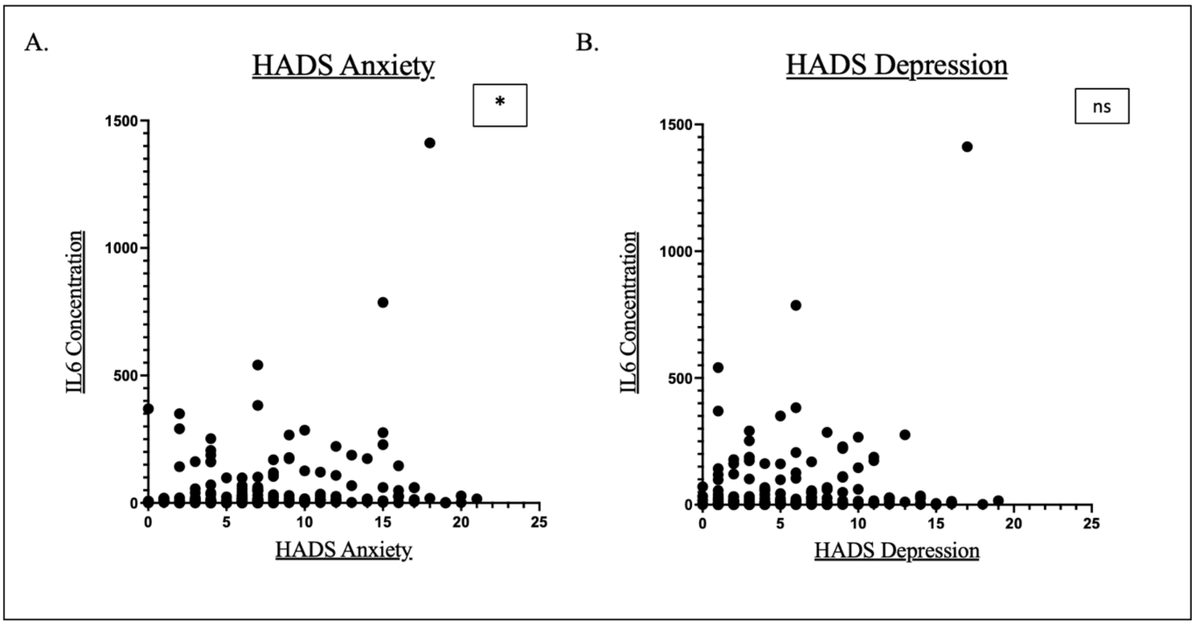

Supplementary Figure 2. Scatterplots showing the correlation between A) IL-6 concentration and HADS anxiety score B) IL-6 concentration and HADS depression score.

The correlation between IL-6 concentration and HADS anxiety had a weak positive correlation coefficient of 0.1645, Fig. 6A). (P = 0.0222).

There is a weak positive correlation between IL-6 concentrations and HADS depression score (correlation coefficient of 0.0712, P = 0.325) (Fig. 6B).

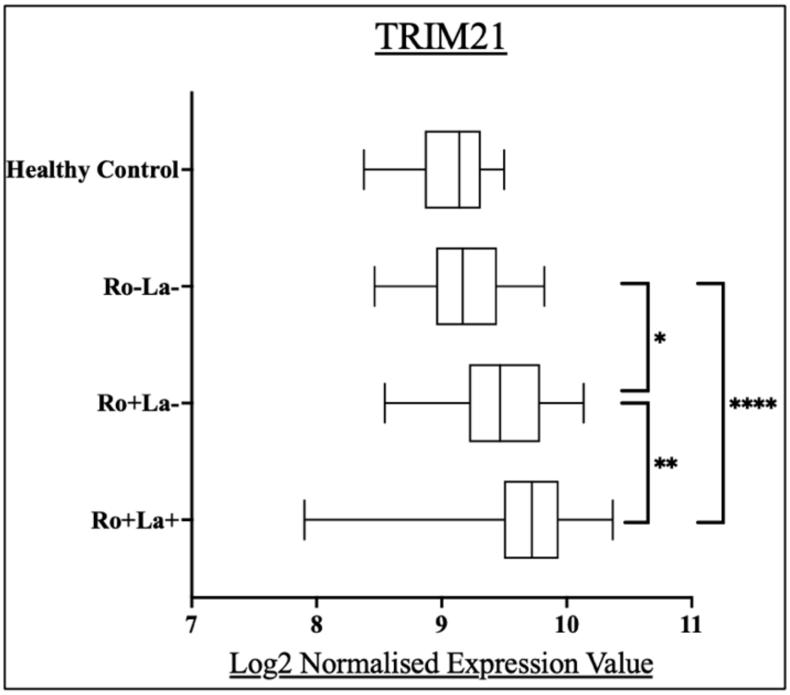

Supplementary Figure 3. Box and whisker plots and associated descriptive statistics tables demonstrating the level of TRIM21 expression in pSS patients divided by serostatus including La positivity compared to healthy controls. Levels of significance were defined as $P \leq 0.05$. Levels of significance are shown by Asterix number: $^{*}P \leq 0.05$, $^{**}P \leq 0.01$, $^{***}P \leq 0.001$, $^{****}P \leq 0.0001$. Graphs were generated in GraphPad prism V.9.

For the group with both Anti-Ro+ and Anti-La+, the median TRIM21 expression was greatest at 9.721. For Anti-Ro+/Anti-La- the median expression was 9.467, whilst patients with both Anti-Ro- and Anti-La- represented a lower median expression at 9.169, comparable to the healthy controls. The TRIM21 expression differences between all autoantibody groups were significant; Anti-Ro+/Anti-La+ versus Anti-Ro+/Anti-La- (P = 0.002), Anti-Ro+/Anti-La+ versus Anti-Ro-/Anti-La- (P<0.0001) and Anti-Ro+/Anti-La- versus Anti-Ro-/Anti-La- (P = 0.0341).
